# Jejunal perforation secondary to blind insertion of peritoneal dialysis catheter: a case report and review of literature

**DOI:** 10.1186/s12882-023-03155-9

**Published:** 2023-04-27

**Authors:** Lucas Jacobs, Mohammed Salaouatchi, Maxime Taghavi, Said Sanoussi, Joelle Nortier, Maria Mesquita

**Affiliations:** 1 Nephrology and Dialysis Department, Brugmann University Hospital, Université Libre de Bruxelles, Brussels, Belgium; 2 Radiology Department, Brugmann University Hospital, Université Libre de Bruxelles, Brussels, Belgium

**Keywords:** Blind peritoneal dialysis catheter insertion, Case report, Peritoneal dialysis, Moncrief-Popovich embedded catheter, Visceral perforation

## Abstract

**Background:**

Peritoneal dialysis (PD) depends upon a functioning and durable access to the peritoneal cavity. Many techniques exist to insert a peritoneal catheter, showing similar outcomes and benefits. Blind percutaneous insertion represents a bedside intervention predominantly performed by nephrologists requiring only local anesthesia, sedation and minimal transcutaneous access. Although current guidelines recommend insertion techniques allowing visualization of the peritoneal cavity, the blind percutaneous approach is still widely used and has been proven safe and effective to bring durable peritoneal dialysis access. Herein, we described a rare case of jejunal perforation secondary to blind PD catheter placement, and conduct a review of the current medical literature describing early bowel perforations secondary to PD catheter placement, gathering descriptions of symptomatology and outcomes and their relations to the insertion technique.

**Clinical presentation:**

We herein describe the case of a 48 year-old patient with a history of appendectomy who suffered from triple jejunal perforation after blind percutaneous insertion and subsequent embedment of his peritoneal catheter. Accurate diagnosis was made 1 month after insertion due to atypical clinical presentation and because physicians had no access to the peritoneal cavity after catheter embedment. After surgical repair and broad-spectrum antibiotics, the patient was switched to HD.

**Conclusion:**

Early catheter-related visceral injury is a rare, yet threatening condition that is almost always causing a switch to hemodialysis or death. Our review highlights that laparoscopic catheter placement might bring better outcomes if perforation occurs, as it allows immediate diagnosis and treatment. On the contrary, catheter embedment may delay clinical diagnosis and therefore bring worse outcomes.

## Background

The success of peritoneal dialysis (PD) as renal replacement therapy depends upon a safe, functional, and durable catheter access to the peritoneal cavity. Furthermore, catheter complications often lead to catheter loss and contribute to technique failure [1]. Several approaches are currently used to implant PD catheters. Namely, blind percutaneous puncture (using a modification of the Seldinger technique), open surgery (“mini-laparotomy”), peritoneoscopy, and surgical laparoscopy. Although each technique has its specific strengths and challenges, it is argued that no single implantation approach produces superior outcomes. Therefore, the 2019 International Society for Peritoneal Dialysis guideline on PD access [1] recommends that the choice of PD catheter insertion method should be based on patient factors, facility resources, and operator expertise. Blind insertion of PD catheter is commonly used and requires neither expensive instruments nor general anesthesia. Although some studies have shown favorable results [2], blind insertion is performed without the identification of the intraperitoneal cavity and is therefore said to be prone to risk of visceral injury compared to other methods. Patients who have undergone previous major abdominal surgery may be at higher risk for visceral injury, nevertheless, bowel perforation is rare. The clinical presentation and outcomes are therefore not well known. The aim of this paper is to describe a rare case of jejunal perforation secondary to blind PD catheter placement, and conduct a review of the current medical literature describing bowel perforations secondary to PD catheter placement, gathering descriptions of symptomatology and outcomes and their relations to the insertion technique.

## Case presentation

We report the case of a 48 year old male patient with end-stage kidney disease (ESKD) secondary to membranous nephropathy. He had a Body Mass Index (BMI) of 28 kg/m2. He underwent an open appendectomy (McBurney’s incision) for non-perforated appendicitis, 20-years before arriving in Belgium. The intervention left a 3 cm scar over McBurney’s point. The patient chose PD after having received complete information about dialysis modalities, and transplantation. Months before, an abdominal computed tomography had been performed and did not show the small intestine adherent to the anterior abdominal wall. Laparoscopic PD catheter placement was temporarily unavailable at the time. The surgical team (specialized in percutaneous PD catheter placements) considered low the probability of adherences secondary to a 20-year old minor surgery that had left a small 3 cm scar on the right flank. Subsequently, as the hernia repair was planned as a small open surgery through a horizontal infra-umbilical 2 cm wide incision, the surgeons opted to place the PD catheter blindly through a left pararectal incision (opposite to McBurney’s point). Benefits and risks were discussed with the patient. Subsequently, embedded PD catheter placement was decided in order to start dialysis within a few weeks. The patient underwent an inguinal hernia repair and concomitant blind insertion of a double-cuffed swan neck catheter for PD according to Moncrief and Popovich technique (i.e. a segment of the catheter was subcutaneously embedded on the abdominal wall) under general endotracheal anesthesia. This procedure was chosen because his clinical condition and biological markers did not require immediate dialysis.

The biology on surgery day was as following: blood urea nitrogen 81 mg/dL; serum creatinine 6.1 mg/dL, corresponding to an estimated glomerular filtration rate of 10 ml/min/1.73m^2^ (as calculated by CKD-EPI [Chronic Kidney Disease Epidemiology Collaboration] equation); serum sodium 135 mEq/L; serum potassium 4.2 mEq/L; serum chloride 99 mEq/L; serum bicarbonate 18 mEq/L; hemoglobin 9.1 g/dL; white blood cell count (WBCC), 10.980/μL; platelet count, 168.000/μL; serum albumin 27 g/L; C-reactive protein (CRP) 10 mg/dl. Following the intervention, the patient presented intermittent vesperal abdominal and lumbar pain, quickly attributed to the recent intervention. He was prescribed painkillers (paracetamol and tramadol) that helped relieve—but not eradicate—the abdominal discomfort. From then on, we steadily observed a growing alteration of his general clinical state and abdominal discomfort. The following days, physical examination showed no signs of acute abdomen but laboratory tests showed progressive increase in inflammatory markers: WBCC 11.000/μL; platelet count 331.000/ μL; serum CRP 139.2 mg/dl. The patient was treated with antibiotics.

**Fig. 1:**
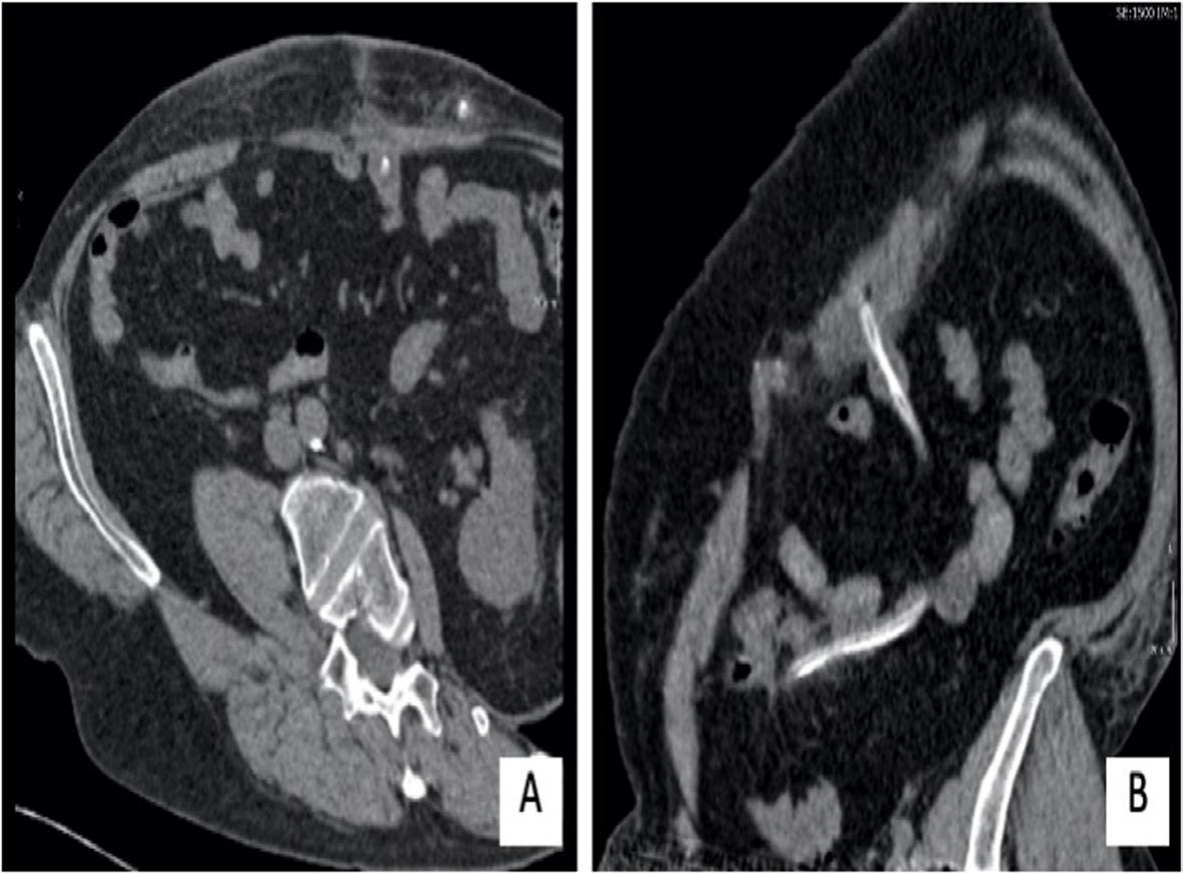
CT Scan of the abdomen: axial oblique (**A**) and coronal oblique (**B**) view showing the intraluminal trajectory of the dialysis catheter

**Fig. 2:**
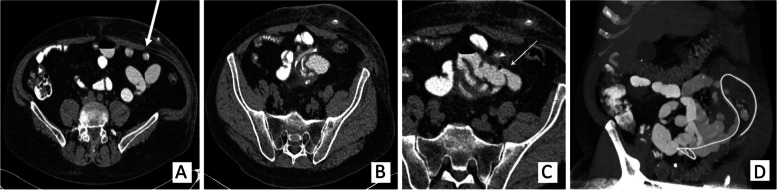
CT scan of the abdomen after oral opacification: axial views confirming the intraluminal trajectory of the catheter (thick arrow) (**A**). Fat infiltration around the distal extremity of the catheter (**B**) and pneumoperitoneum with extraluminal gas (thin arrow) (**C**). Coronal maximal intensity projection view showing the course of the catheter (**D**)

Finally, 1 month after PD catheter placement, the patient presented to the emergency department due to deterioration of general condition and aggravation of abdominal pain. On clinical examination, our patient was afebrile, with diffuse pain on abdominal palpation, presence of peristalsis but significant cellulitis of the entire abdominal wall and tunnelitis of the embedded PD catheter. An abdominal computed tomography (CT) scan showed the PD catheter in the lumen of the small intestine through several bowel perforations (Figs. 1a-b and 2a-b-c) along with the presence of two microbubbles of extravisceral gas. A Coronal projection view even showed the course of the catheter inside the lumen of the jejunum (Fig. 2d).

Surgical intervention was planned urgently. Laparoscopic surgery revealed the jejunum adhering to the anterior wall with the intraluminal dialysis catheter. Following the small intestine, the surgeons came across a magma containing several adherent intestinal loops. Adhesiolysis was performed, the abdominal exploration showed the peritoneal catheter perforating a first intestinal loop to enter a completely perforated second. The catheter came out a second time, reentering a jejunal loop nearby. There were ultimately several perforations: respectively at 140 cm, 150 cm, 180 cm and 2 m from the angle of Treitz. The patient underwent 48 cm of segmental jejunal resection and the removal of the PD catheter. He recovered from his abdominal intervention but had to continue with hemodialysis (HD) while his choice for renal replacement therapy was initially PD. He benefited from a tunneled right internal jugular catheter and started on chronic intermittent HD while receiving 14 days of intermittent piperacillin-tazobactam antibiotics.

## Discussion

Several techniques are established regarding PD catheter implantation in the peritoneal cavity. Namely, open surgery, basic or advanced laparoscopy, peritoneoscopy, and percutaneous puncture (blind or with image guidance). However, the optimal way of establishing access for peritoneal dialysis remains disputed. Many studies have compared the different insertion techniques, historically showing similar outcomes and complication rates, leaving the choice of insertion approach controversial, and largely dependent on local medical expertise and hospital facilities. It is worth noticing, however, that a recent meta-analysis demonstrated significantly superior outcomes for advanced laparoscopy over open surgery or basic laparoscopy with regard to catheter tip migration, flow obstruction, and catheter survival as it allowed to perform adjunctive procedures as omentopexy, adhesiolysis, or epiploectomy, therefore facilitating laparoscopic visualization of the peritoneal cavity for adhesions and hernias [3].

Percutaneous catheter insertion, represents a bedside intervention predominantly performed by nephrologists requiring only local anesthesia, sedation and minimal transcutaneous access. In addition to decreased patient hardship, the technique also entails socioeconomic gain as it limits the use of the surgical theater. Notably, two meta-analysis of prospective and retrospective cohort studies have shown that the percutaneous access is safe, effective, and offers satisfactory and equivalent outcomes in selected patients (such as no previous abdominal surgery, BMI < 28 kg/m2) with regards to infectious and mechanical complications in comparison to surgical techniques [4, 5].

Unfortunately, catheter-related complications still exist and may lead to technical failure, which directly affect the long-term catheter survival and can ultimately lead to permanent conversion to hemodialysis in up to 20% of patients [6]. Early complications are those occurring in the perioperative period. Among the most feared early complications, visceral injuries are reported in both the open and closed insertion techniques, and the symptomatology is said to be sometimes misleading [7]. The 2019 ISPD guidelines on PD access recommend a yearly visceral perforation rate < 1% due to PD catheter insertion given that fecal peritonitis is associated with significant morbidity and mortality [8]. Techniques allowing direct vision, such as laparoscopic or open surgical insertion, are therefore primarily recommended for more complicated patients including those with a previous laparotomy incision, previous severe or recurrent peritonitis, morbid obesity, or distorted anatomy [1]. It implies that visceral perforation would be more frequent in blind percutaneous PD catheter insertion. It is noteworthy that the < 1% visceral perforation recommended rate is based on incidence rates of 4 studies reviewing respectively a different approach of catheter insertion [9–12].

Herein, we performed a systematic search of the medical literature on early visceral injury secondary to PD catheter placement using the PubMed database, Google Scholar, and Cochrane library for related published articles. We excluded cases of clear delayed perforation (symptoms starting 10 to 15 days after insertion) as it would arguably not be secondary to the insertion procedure, we also excluded pediatric cases. We gathered every published case (ours included) of early bowel perforation secondary to PD catheter insertion (every insertion approach included), further describing symptomatology, diagnostic delays, treatments and outcomes. To our knowledge, correlating bowel perforation-related outcomes and the insertion technique used has not been studied yet. In the current medical literature, we found 7 articles (Table 1): 5 case reports [13–17] and two descriptive retrospective case studies [18, 19]. We have considered “recent” articles if published after the year 2000 (4 articles), and older if published before the year 2000 (3 articles).

**Table 1:** Patient’s characteristics in the literature

**Authors**	**Gender (Age)**	**History of surgery or peritonitis**	**Insertion technique and point**	**Diagnosis delay**	**Clinical presentation**	**Treatment**	**Outcome**
**Jacobs. 2022**	M (48)	Appendectomy	Blind percutaneous insertion; paramedian	30 days	Abdominal discomfort, slow deterioration of general condition, sepsis	Surgical sutures; IV piperacillin- tazobactam (total 14 days)	HD
**Raksasuk. 2020 **[13]	M (78)	None	Blind percutaneous insertion; subumbilical	4–12 h	Yellow dialysate (4 h)—High grade fever (12 h)	Surgical sutures; IV meropenem	HD
**Shima. 2018** [14]	M (78)	Unknown	Unknown	Unknown	Watery diarrhea, Relapsing peritonitis	Surgical sutures; IV ceftriaxone, switched to IV meropenem	HD
**Abreo. 2016** [15]	M (69)	Recent laparotomy	Fluoroscopic percutaneous insertion; subumbilical	9 h	Acute abdomen, vomiting, hypotension	Broad spectrum antibiotics	Death
**Asif. 2003** [18]	W (55)	None	Peritoneoscopic; subumbilical	None	Per-operative notice of foul-smelling gas	IV vancomycin, gentamicin, and metronidazole. Oral relay with ampicillin, metronidazole, and gentamicin (total 10 days)	HD
	W (38)	None	Peritoneoscopic; subumbilical	None	Per-operative notice of foul-smelling gas	IV vancomycin, gentamicin, and metronidazole. Oral relay with ampicillin, metronidazole, and gentamicin (total 10 days)	PD
	M (55)	Recent peritonitis	Peritoneoscopic; subumbilical	None	Per-operative visualization of intrabowel cannula; per-operative notice of foul-smelling gas	IV vancomycin, gentamicin, and metronidazole. Oral relay with ampicillin, metronidazole, and gentamicin (total 10 days)	HD
	W (54)	None	Peritoneoscopic; subumbilical	None	Per-operative notice of foul-smelling gas	IV ticarcillin disodium/clavulanate potassium and metronidazole. Oral relay with gatifloxacin and metronidazole (total 14 days)	Off-dialysis
	M (72)	None	Peritoneoscopic; subumbilical	None	Per-operative notice of foul-smelling gas; high grade fever and acute abdomen the next day	IV vancomycin, gentamicin, and metronidazole	HD
**Rigolosi. 1969** [16]	W (79)	Unknown	Blind percutaneous; subumbilical	Few hours	Failed dialysis fluid drainage; malodorous fluid return; watery diarrhea	Unknown	Death
**Simkin. 1968** [19]	M (19)	Intra-abdominal injury	Blind percutaneous; right or left iliac fossa	None	Feculent returning fluid	IV Chloramphenicol; Laparotomy repair	Death
	M (47)	None	Blind percutaneous; right or left iliac fossa	None	Failed dialysis fluid drainage and watery diarrhea	IV Chloramphenicol	Death
	M (46)	Several abdominal surgeries	Blind percutaneous; right or left iliac fossa	Unknown	Feculent returning fluid, rectal dialysis fluid evacuation, vomiting	IP Chloramphenicol	PD
	M (34)	None	Blind percutaneous; right or left iliac fossa	2 h	Failed dialysis fluid drainage, acute abdomen, feculent returning fluid	IP Chloramphenicol; Laparotomy repair	HD
	W (56)	Several abdominal surgeries	Blind percutaneous; right or left iliac fossa	5 h	Altered general state, acute abdomen	IP Chloramphenicol	Death
**Krebs. 1966** [17]	M (54)	Temporary PD catheter removed days before	Blind percutaneous; right iliac fossa	Few hours	Failed dialysis fluid drainage; rectal dialysis fluid evacuation	Laparotomy repair	Death

*M* Man, *W* Woman, *HD* Hemodialysis, *PD* Peritoneal dialysis

We found 4 articles describing bowel perforations secondary to blind percutaneous punctures, 1 secondary to fluoroscopic percutaneous insertion, 1 secondary to peritoneoscopic insertion, and 1 unknown. In total, 16 patients are reported, out of whom 5 had a history of peritonitis or abdominal surgery.

Peritoneoscopic catheter insertion was always characterized by immediate diagnosis, as injuries were diagnosed during the intervention. However, after percutaneous puncture (blind or fluoroscopic) diagnosis delays vary, and bowel perforations are then diagnosed through clinical follow-up.

Symptoms include: minor abdominal signs but yellow dialysate effluent and high grade fever [13]; watery diarrhea and relapsing peritonitis [14]; acute abdomen, hypotension, and vomiting [15]. Simkin and Wright’s [19] described bowel perforations revealed by fecal returning dialysate (3 out of 5 (3/5) patients), failed fluid drainage (2/5), acute abdomen (2/5), watery diarrhea (1/5), vomiting (1/5) and even rectal evacuation of dialysis fluid (1/5). Two other older articles report two patients respectively suffering from failed dialysis fluid drainage, malodorous fluid return and watery diarrhea [16], and failed dialysis drainage along with rectal dialysis fluid evacuation [17].

In recent reports, each patient was treated with broad-spectrum antibiotics. Antibiotics were generally intravenous, rarely given intraperitoneally as PD was often quickly abandoned in favor of HD. Older studies as Rigolosi’s and Krebs’ do not mention antibiotics while patients in Simkin and Wright’s study were treated with IV or IP chloramphenicol. In recent studies, surgical sutures were performed in all but 1 patient following percutaneous placement (see Table 1). It was not specified if perforations after peritoneoscopy resulted in per-operative suture of injured bowel. In older studies, surgical repair was performed in 3 out of 7 patients, while Rigolosi’s study did not specify.

Out of the 3 recent cases (ours included) of percutaneous insertion, no patient could carry on with PD: 2 patients were shifted to HD, one patient died [13, 15]. In Rigolosi and Krebs’ cases of percutaneous insertions, both patients died secondary to bowel perforation; in Simkin and Wright’s cases of percutaneous insertions, 3 out of 5 patients died, 1 was shifted to HD, 1 continued PD, although the 1960’s medical care has poor representability nowadays. Peritoneoscopic placement resulted in a per-operative diagnosis of visceral injury; consecutively, no patient died but 3 out of 4 patients would be switched to HD.

Our patient presented immediate yet not flagrant abdominal discomfort. He had no fever, nor vomiting or diarrhea and was hemodynamically stable, although he suffered from jejunal perforation due to the PD catheter. He gradually developed his abdominal symptoms until a flagrant acute abdomen 1 month after hernia repair, subsequent blind catheter insertion and embedment. The 2019 ISPD guidelines suggest to consider concomitant hernia repair and catheter embedment (Moncrief-Popovich technique). Catheter embedment being compatible with any of the implantation approaches using any catheter device, percutaneous blind insertion was chosen [20]. There is effectively no data linking the Moncrief–Popovich technique to bowel perforation.

Our literature review has shown that visceral injury due to the peritoneal catheter has varying symptoms and that even a triple jejunal perforation can initially be paucisymptomatic. Catheter embedment is said to be less recommended in patients who have had previous major abdominal surgery or peritonitis, because adhesiolysis during the implantation procedure increases the probability of subsequent catheter obstruction by recurrence of adhesions. It should be noted that risk factors for early visceral perforation due to PD catheter implantation, and risk factors for catheter dysfunction after catheter embedment are identical. In 1968, Simkin and Wright put forward 3 criteria for the diagnosis of bowel perforation occurring during peritoneal dialysis: (i) retention of the dialysate in the abdomen; (ii) cloudy, malodorous, or frankly feculent fluid return; and (iii) watery diarrhea [19]. Although feculent dialysis fluid or rectal fluid evacuation are not frequent nowadays, we would still argue that having embedded the PD catheter resulted in a prolonged diagnosis delay as 2 diagnostic criteria out of 3 were unavailable. Had we been able to perform dialysis fluid exchanges, we could have possibly noticed a discolored returning fluid or failed fluid drainage, sent a fluid sample to bacterial culture, or proceeded to fluoroscopic control. However, retrospectively, ultrasound control of PD catheter trajectory or abdominal CT scan should have been used faster in order to look for early complications.

Our case report shows that even in the case of an old minor surgery, leaving a small scar on the side opposite the dialysis catheter implantation, visceral adhesions to the abdominal wall can be major and cause a significant risk of bowel perforation. Thus, no exception should be made to the rule of favouring implantation techniques that allow visualization of the catheter in the abdominal cavity.

## Conclusion

Visceral perforation due to PD catheter brings generally nonspecific symptoms. Furthermore, the outcomes following bowel perforation in peritoneal dialysis depends largely on diagnosis delay. Unfortunately, even with prompt diagnosis and treatment of visceral injury, patients are generally switched to hemodialysis. However, immediate diagnosis results in lower mortality given that treatment is urgently initiated. The present study therefore adheres to the 2019 ISPD guidelines recommending insertion techniques allowing direct visualization of the peritoneal cavity. Although the current medical literature does not seem to prove a higher incidence rate of visceral trauma using the blind percutaneous insertion technique in selected patients, it would certainly seem to delay the diagnosis of bowel injury and therefore bring worse outcomes. Blind insertion and subsequent embedment of PD catheter in patients with history of abdominal surgery or peritonitis is at risk of visceral perforation and increased diagnostic delay.

## Data Availability

Not applicable.

## References

[1] Crabtree JH, Shrestha BM, Chow KM, Figueiredo AE, Povlsen JV, Wilkie M (2019). Creating and maintaining optimal peritoneal dialysis access in the adult patient: 2019 update. Perit Dial Int.

[2] Kang SH, Do JY, Cho KH, Park JW, Yoon KW (2012). Blind peritoneal catheter placement with a Tenckhoff trocar by nephrologists: a single-center experience. Nephrology (Carlton).

[3] Shrestha BM, Shrestha D, Kumar A, Shrestha A, Boyes SA, Wilkie ME (2018). Advanced laparoscopic peritoneal dialysis catheter insertion: systematic review and meta-analysis. Perit Dial Int.

[4] Boujelbane L, Fu N, Chapla K, Melnick D, Redfield RR, Waheed S (2015). Percutaneous versus surgical insertion of PD catheters in dialysis patients: a meta-analysis. J Vasc Access.

[5] Tullavardhana T, Akranurakkul P, Ungkitphaiboon W, Songtish D (2016). Surgical versus percutaneous techniques for peritoneal dialysis catheter placement: a meta-analysis of the outcomes. Ann Med Surg (Lond).

[6] Maio R, Figueiredo N, Costa P (2008). Laparoscopic placement of Tenckhoff catheters for peritoneal dialysis: a safe, effective, and reproducible procedure. Perit Dial Int.

[7] Oza-Gajera BP, Abdel-Aal AK, Almehmi A (2022). Complications of percutaneous peritoneal dialysis catheter. Semin Intervent Radiol.

[8] Wakeen MJ, Zimmerman SW, Bidwell D (1994). Viscus perforation in peritoneal dialysis patients: diagnosis and outcome. Perit Dial Int.

[9] Zappacosta AR, Perras ST, Closkey GM (1991). Seldinger technique for Tenckhoff catheter placement. ASAIO Trans.

[10] Gadallah MF, Pervez A, el-Shahawy MA, Sorrells D, Zibari G, McDonald J (1999). Peritoneoscopic versus surgical placement of peritoneal dialysis catheters: a prospective randomized study on outcome. Am J Kidney Dis.

[11] Crabtree JH, Fishman A (2005). A laparoscopic method for optimal peritoneal dialysis access. Am Surg.

[12] Dunne N, Booth MI, Dehn TCB (2011). Establishing pneumoperitoneum: Verres or Hasson? The debate continues. Ann R Coll Surg Engl.

[13] Raksasuk S, Taweerautchana W, Srithongkul T (2020). Jejunal perforation during peritoneal dialysis catheter placement: a case report. Ann Med Surg (Lond).

[14] Shima H, Mizoguchi S, Morine Y, Tashiro M, Okada K, Minakuchi J (2018). Intestinal perforation by a peritoneal dialysis catheter in which fungal peritonitis led to diagnosis: a rare case report. CEN Case Rep.

[15] Abreo K, Sequeira A (2016). Bowel perforation during peritoneal dialysis catheter placement. Am J Kidney Dis.

[16] Rigolosi RS, Maher JF, Schreiner GE (1969). Intestinal perforation during peritoneal dialysis. Ann Intern Med.

[17] Krebs RA, Burtis BB (1966). Bowel perforation. A complication of peritoneal dialysis using a permanent peritoneal cannula. JAMA.

[18] Asif A, Byers P, Vieira CF, Merrill D, Gadalean F, Bourgoignie JJ (2003). Peritoneoscopic placement of peritoneal dialysis catheter and bowel perforation: experience of an interventional nephrology program. Am J Kidney Dis.

[19] Simkin EP, Wright FK (1968). Perforating injuries of the bowel complicating peritoneal catheter insertion. Lancet.

[20] Crabtree JH, Burchette RJ (2009). Effect of prior abdominal surgery, peritonitis, and adhesions on catheter function and long-term outcome on peritoneal dialysis. Am Surg.

